# Supplementary material for: Production and Evaluation of Fluorophore-Doped Polymer Substrates to Screen for Plastic-Degrading Enzymes
Source: ACS Sustain Chem Eng. 2026 Mar 25;14(13):6485–94. doi: 10.1021/acssuschemeng.6c00030 (PMC13058888; doi:10.1021/acssuschemeng.6c00030)
Supplement: Supplementary file 1 [file sc6c00030_si_001.pdf]

Production and evaluation of fluorophore-doped polymer substrates to screen for plastic-degrading enzymes

Anton A. Stepnov, [1] Brenna Norton-Baker, [2,3] Esteban Lopez-Tavera, [1] Ravindra R. Chowreddy, [4] Vincent G. H. Eijsink, [1] Gregg T. Beckham, [2,3] Gustav Vaaje-Kolstad[1*]

[1] Faculty of Chemistry, Biotechnology and Food Science, NMBU - Norwegian University of Life Sciences, Ås, 1433 Norway

[2] Renewable Resources and Enabling Sciences Center, National Laboratory of the Rockies, Golden, CO, 80401 USA

[3] BOTTLE Consortium, Golden, CO, 80401 USA

[4] Norner Research AS, Porsgrunn, NO-3920 Norway

* - corresponding author: gustav.vaaje-kolstad@nmbu.no

Supporting Information: 22 pages, 15 figures, 1 table

Table S1. Melting temperatures and crystallinity of the materials used in this study. The data were acquired in duplicates using differential scanning calorimetry (DSC).

**Material**	**Melting temperature, °C**	**Degree of crystallinity, %**
PET	219.7 ± 2.1	33.3 ± 0.7
PET-R6G	231.9 ± 0	4.8 ± 0.9
HDPE	131.4 ± 0.4	62.9 ± 0.2
HDPE-R6G	132.8 ± 0.6	60.6 ± 0.4
PA6	216.6 ± 0.9	29.2 ± 0.4
PA6-R6G	215.9 ± 0.2	30.8 ± 0.4
PA66	260.4 ± 0.1	27.6 ± 0.6
PA66-R6G	261.4 ± 0.5	26.4 ± 0.1

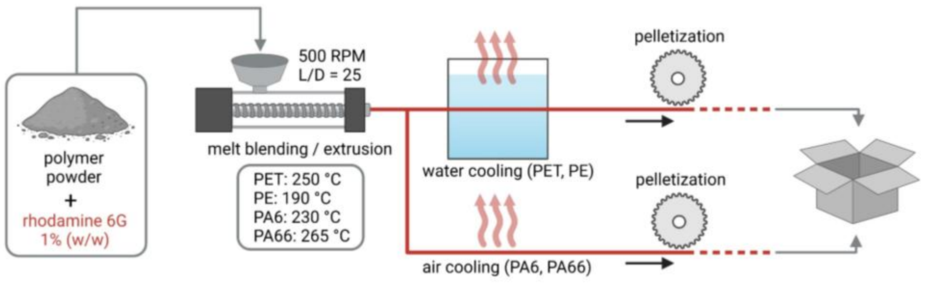

Figure S1. Schematic representation of the melt blending process used to create fluorophore-doped materials in this study. The figure was created with BioRender (www.biorender.com).

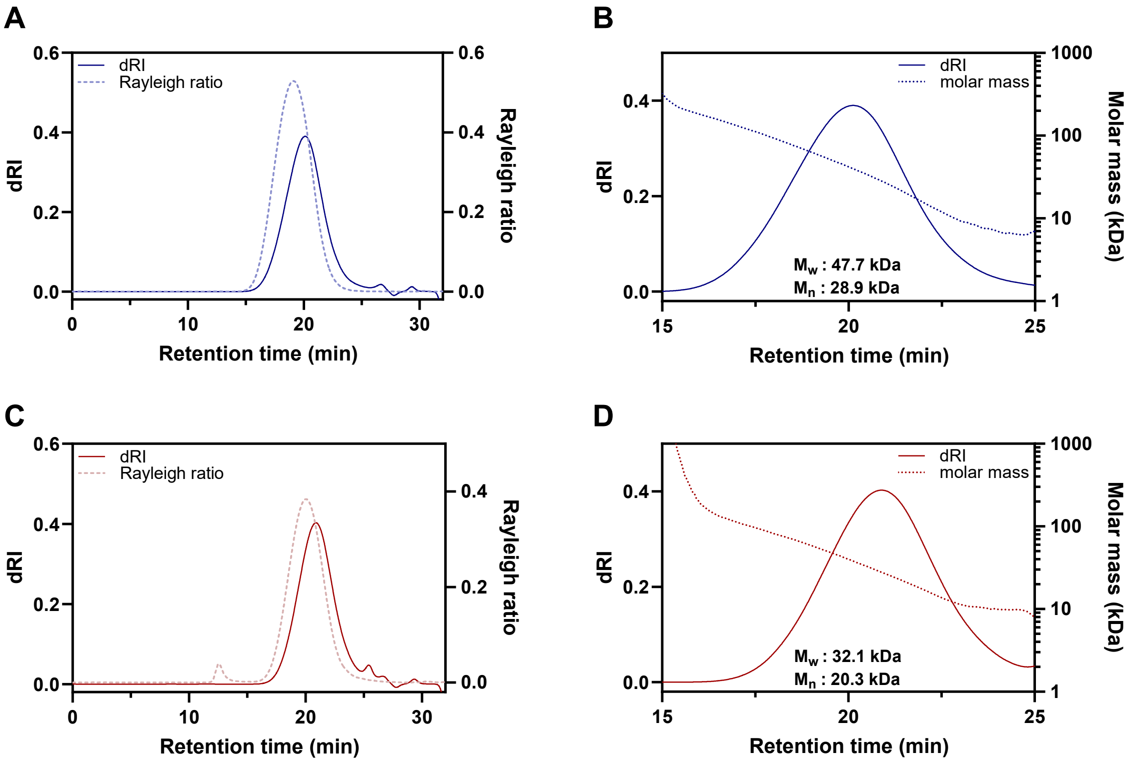

Figure S2. SEC-MALS analysis of PET before and after melt blending with rhodamine 6G. The chromatograms in (**A,B**) and (**C,D**) are for the pristine and fluorophore-doped plastics, respectively. The normalized signals from a differential refractive index detector and a MALS (multi-angle light scattering) detector are shown in **(A,C)** whereas the calculated molar mass values are presented in **(B,D)**. M_w, weight average molar mass; M_n, number average molar mass. The analyses were performed once. Source data are provided as a Source Data file.

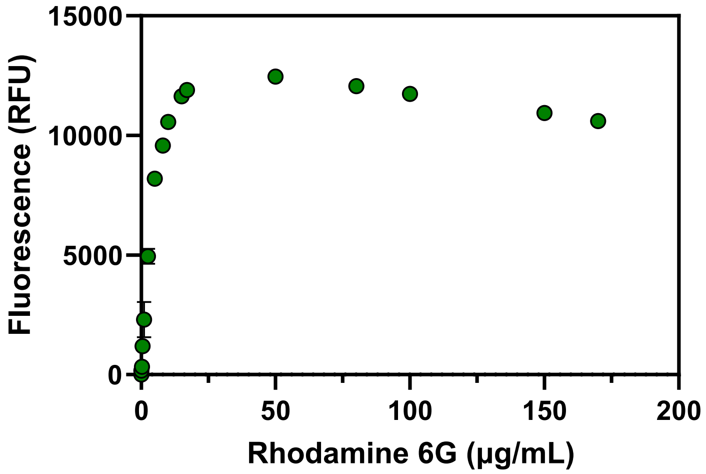

Figure S3. Fluorescence of standard solutions of rhodamine 6G. The fluorescence data was obtained using solutions with various concentrations of free rhodamine 6G ($\lambda_{ex/em}$ = 530/552 nm) in 50 mM Tris-HCl buffer, pH 8.0. Error bars indicate standard deviations between triplicate measurements and are in most cases hidden behind the data markers. Note that increasing the fluorophore concentration beyond 50 µg/mL results in a decrease in fluorescence. Source data are provided as a Source Data file.

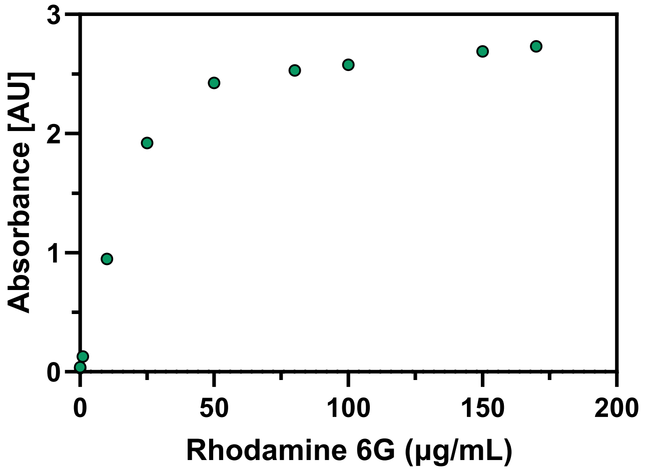

Figure S4. Light absorbance of rhodamine 6G standard solutions. The data was acquired at 530 nm in a 96-well microtiter plate with a <1 cm optical path. 200 µL samples were prepared in triplicates in 50 mM Tris-HCl, pH 8.0. Error bars indicating standard deviations are hidden behind the data markers. Source data are provided as a Source Data file.

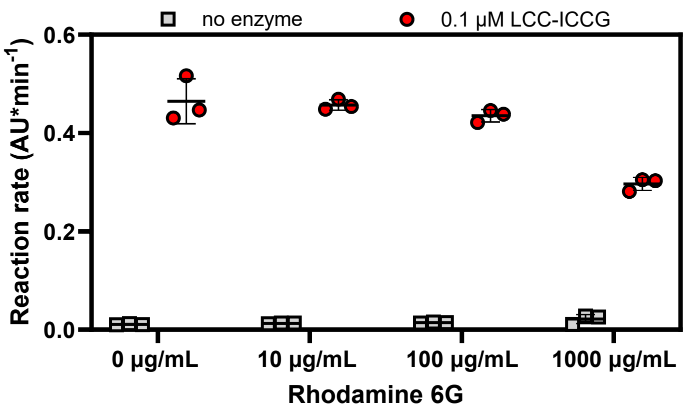

Figure S5. Effect of rhodamine 6G on the hydrolysis of 4-nitrophenyl acetate by LCC-ICCG. The reactions were carried out at room temperature in 50 mM Tris-HCl, pH 8.0, using 1 mM substrate and 0.1 µM enzyme. Error bars indicate standard deviations between triplicate measurements (all individual data is shown), whereas horizontal lines denote average values. Reaction progress was followed by monitoring the absorbance of 4-nitrophenol at 400 nm. The reaction rates were determined using the linear (initial) parts of the progress curves. Source data are provided as a Source Data file.

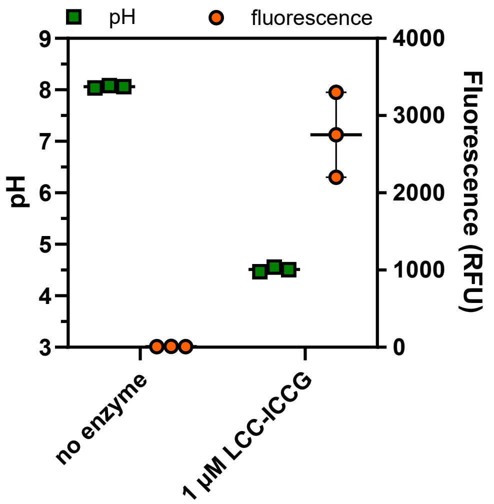

Figure S6. Acidification of reaction mixtures during enzymatic hydrolysis of PET-R6G. The reactions were carried out for 96 h at 65 °C in 50 mM Tris-HCl, pH 8.0 using one PET-R6G pellet (~17 mg; 1.7% w/v solids loading) and 1 µM (28.8 µg) LCC-ICCG (1.7 mg enzyme/g PET). Fluorescence was recorded after diluting the sample ten times with the reaction buffer. Error bars indicate standard deviations between replicates (*n* = 3) whereas horizontal lines denote average values. Source data are provided as a Source Data file.

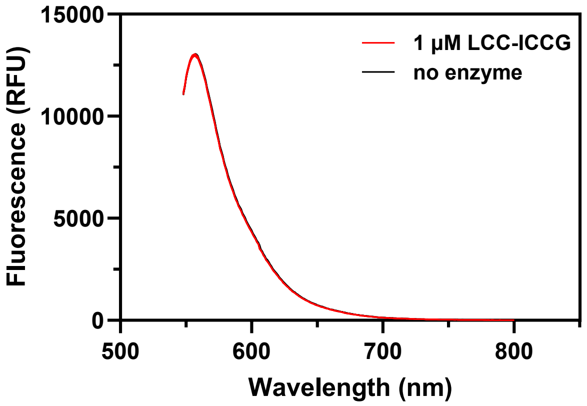

Figure S7. Emission spectrum of free rhodamine 6G after incubation in the presence or absence of LCC-ICCG. The reactions were carried out at 37 °C in 50 mM Tris-HCl buffer, pH 8.0 using 100 μg/mL rhodamine 6G and 1 μM enzyme. Triplicate experiments were conducted, and the spectral data were acquired at 530 nm excitation wavelength. Six individual overlapping traces are shown. Source data are provided as a Source Data file.

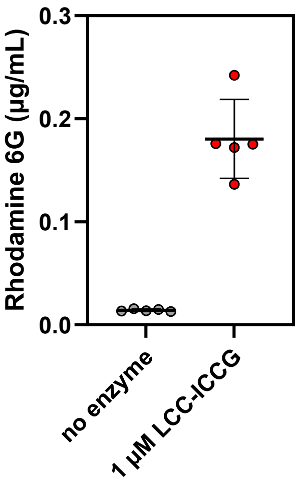

Figure S8. Enzymatic hydrolysis of PET-R6G at 37 °C. The 1 mL reaction mixtures containing 50 mM Tris-HCl buffer, pH 8.0, one substrate pellet (≈17 mg; 1.7% w/v solids loading) and 1 μM (28.8 μg) LCC-ICCG (1.7 mg enzyme/g PET) were incubated at 37 °C, 500 RPM for 19 h. 200 μL aliquots were taken from reaction mixtures and transferred to a 96-well microtiter plate. Fluorescence was measured immediately ($\lambda_{ex/em}$ = 530/552 nm) and the concentration of rhodamine 6G was determined according to a standard curve. Error bars indicate standard deviations between replicate measurements (*n = 5*) whereas horizontal lines denote average values. All individual data points are shown. Source data are provided as a Source Data file.

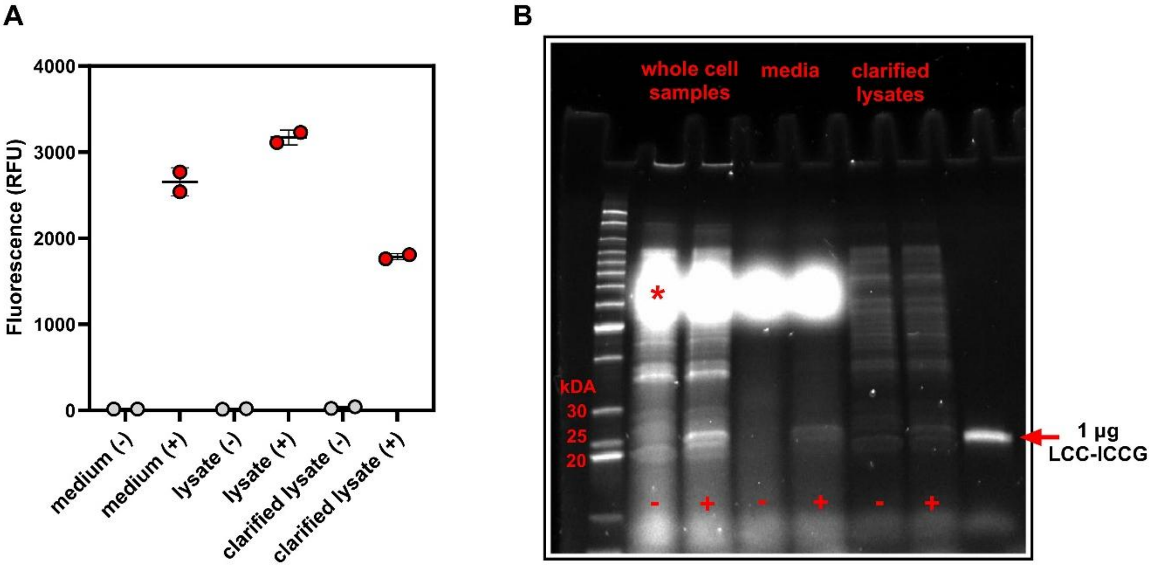

Figure S9. Detecting PETase activity in crude biological samples. (A) Degradation of PET-R6G observed in experiments with *E. coli* lysates and cell-free culture media and **(B)** results of SDS-PAGE analysis of these preparations compared to whole cell samples (i.e., total culture prior to lysis). LCC-ICCG-containing samples are marked with "+", whereas negative control samples are labeled with "-". The dilution factor was the same across all experimental conditions, meaning that the band intensities reflect the true differences in protein abundance. The image color was inversed to improve visual clarity. The bright high molecular mass bands (annotated with "*") originate from medium compounds interfering with the stain-free protein detection system. The PET-R6G degradation experiments were carried out in duplicates for 60 hours at 60 °C, 500 RPM, in 0.5 mL of 50 mM Tris-HCl, pH 8.0 (1 pellet per reaction). Error bars indicate standard deviations; horizontal lines denote average values. Source data are provided as a Source Data file.

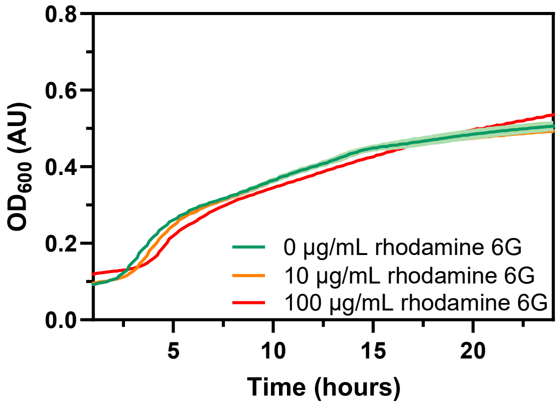

Figure S10. Effect of rhodamine 6G on growth of *E. coli*. Growth curves of BL21 Star (DE3) cells incubated in LB medium at 37 °C in the presence of 0, 10, or 100 µg/mL rhodamine 6G. Error envelopes (visible for the green trace only) indicate standard deviations between triplicate measurements. The experiment was conducted in a microtiter plate with a <1 cm optical path. Source data are provided as a Source Data file.

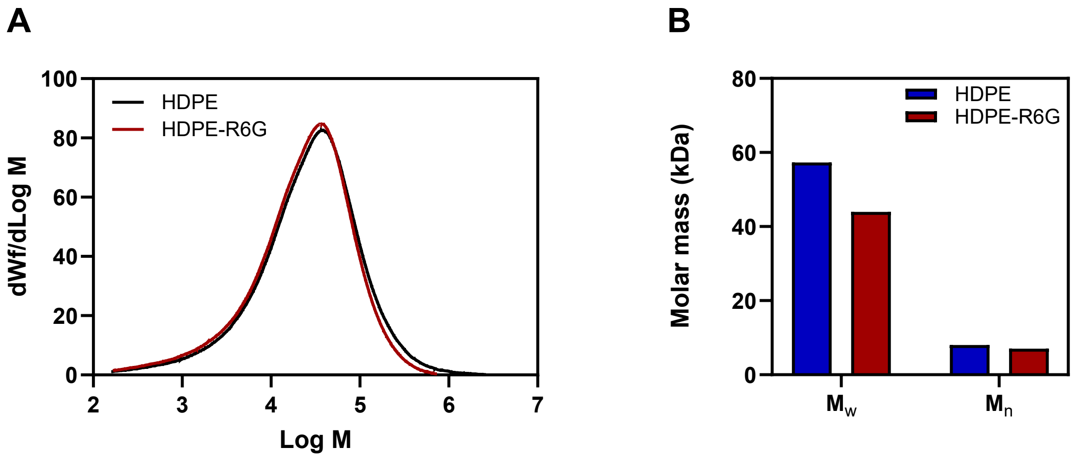

Figure S11. SEC analysis of HDPE before and after melt blending with rhodamine 6G. (A) Differential molar mass distribution curves and **(B)** weight average (M_w) and number average (M_n) molar mass. The analyses were performed once. Source data are provided as a Source Data file.

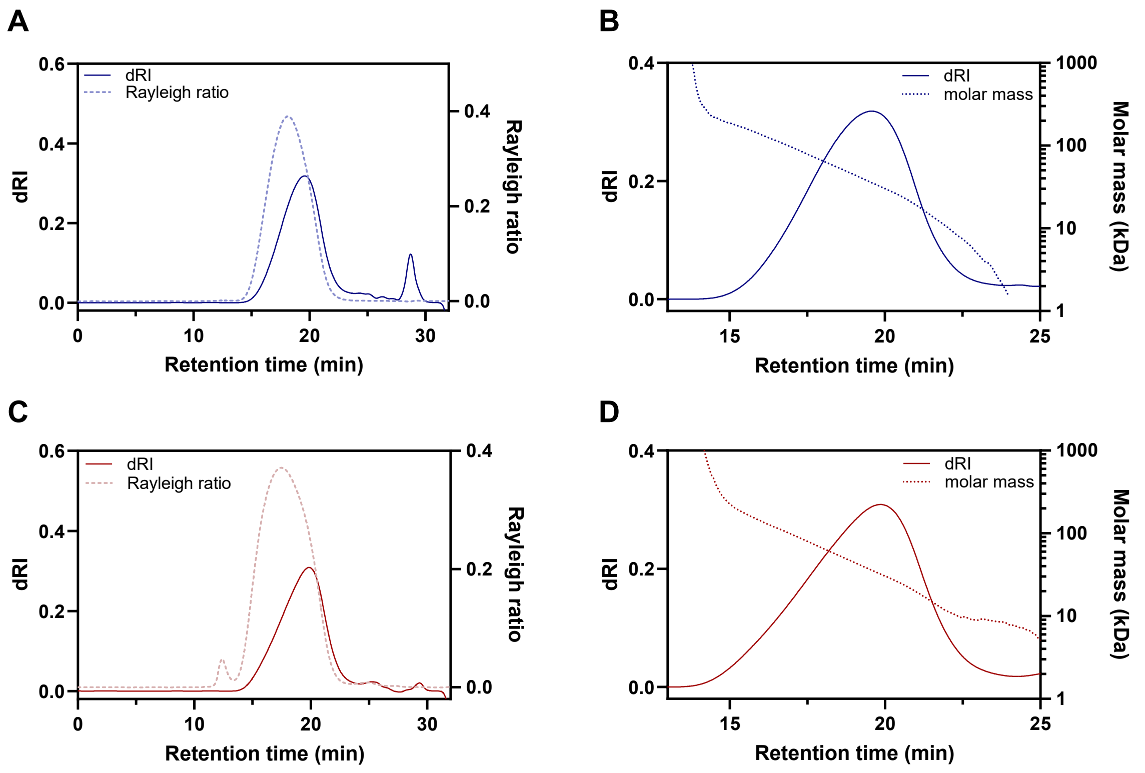

Figure S12. SEC-MALS analysis of nylon 6 before and after melt blending with rhodamine 6G. The chromatograms in (**A,B**) and (**C,D**) are for the pristine and fluorophore-doped plastics, respectively. The normalized signals from a differential refractive index detector and a MALS (multi-angle light scattering) detector are shown in **(A,C)** whereas the calculated molar mass values are presented in **(B,D)**. Note that precise determination of the weight average and number average molar mass was not possible due to a pronounced multimodality and tailing of the polymer peak. The analyses were performed once. Source data are provided as a Source Data file.

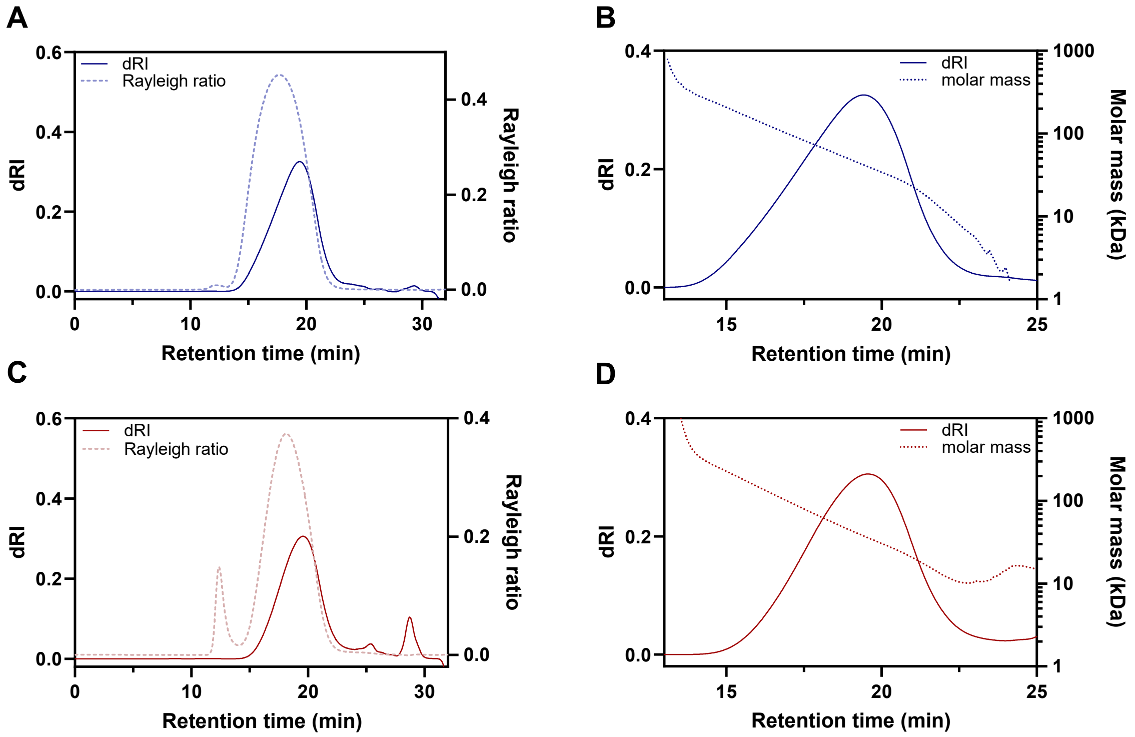

Figure S13. SEC analysis of nylon 6,6 before and after melt blending with rhodamine 6G. The chromatograms in (**A,B**) and (**C,D**) are for the pristine and fluorophore-doped plastics, respectively. The normalized signals from a differential refractive index detector and a MALS (multi-angle light scattering) detector are shown in **(A,C)** whereas the calculated molar mass values are presented in **(B,D)**. Note that precise determination of the weight average and number average molar mass was not possible due to a pronounced multimodality and tailing of the polymer peak. The analyses were performed once. Source data are provided as a Source Data file.

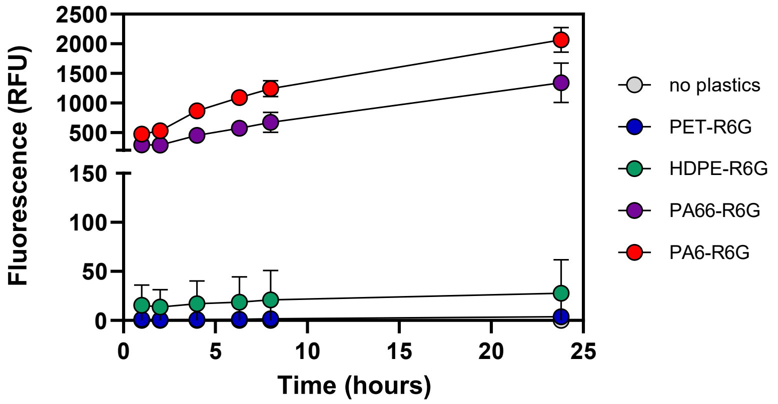

Figure S14. Leaching of rhodamine 6G from various blended plastics. The curves show the increase in fluorescence observed during the incubation of blended materials in 1 mL of 50 mM Tris-HCl, pH 8.0, at 65 °C, 1000 RPM (1 pellet per experiment). Error bars indicate standard deviations between triplicate measurements. Note that the fluorescence values ($\lambda_{ex/em}$ = 530/552 nm) were obtained after 10-fold dilution with buffer. All rhodamine containing materials were prepared at 1% (w/w) rhodamine 6G loading, but the actual loading of the resulting materials may have been lower. Source data are provided as a Source Data file.

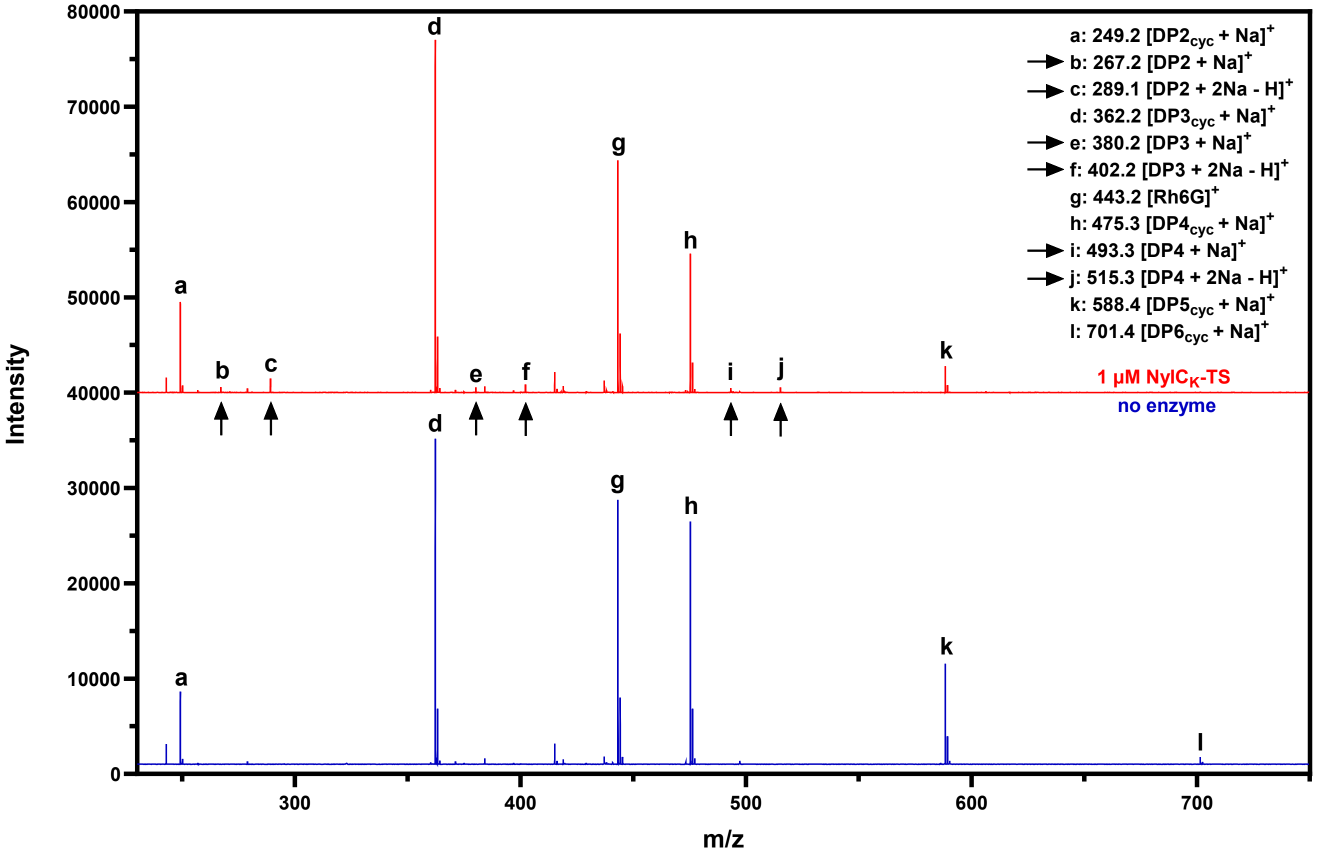

Figure S15. NylC$_K$-TS activity on PA6-R6G. The MALDI-ToF MS spectra were obtained after 265 hours of incubation of PA6-R6G (~11 mg; 1.1 % w/v solids loading) in the presence or absence of 1 µM (39 µg; 3.5 mg enzyme/g nylon) NylC$_K$-TS in 1 ml of 50 mM sodium phosphate buffer, pH 7.4, supplied with 150 mM NaCl. The experiments were carried out at 65°C, 500 RPM. Cyclic and linear oligomers of nylon-6 were observed as sodium adducts, whereas rhodamine 6G was detected as a free cation. The linear products released upon enzyme addition are marked with arrows. Note that cyclic oligomers with a degree of polymerization of 2-6 ("DP2-6$_{cyc}$") were present in the control reaction lacking the nylonase, indicating substrate contamination (see main text for more discussion). The mass spectra indicate depletion of cyclic hexamer ("l") and cyclic pentamer ("k") in the reaction with enzyme, suggesting that some of the linear products were generated as a result of nylonase acting on these contaminants rather than on the polymeric nylon-6 substrate. Source data are provided as a Source Data file.

Supplementary methods

Chemicals and materials

Chemicals were sourced from Sigma-Aldrich (St. Louis, MO, USA) unless specified otherwise. Low-melting point PET (CumaPET L04 100) was obtained from DuFor Resins BV (Zevenaar, the Netherlands). Nylon 6 and Nylon 6,6 resins (Technyl® Shape C 402M NC; Technyl® Safe A 402FC NC, respectively) were sourced from DOMO Chemicals (Gent, Belgium). High density polyethylene (grade SHA7260) was sourced from Braskem Europe (Wesseling, Germany). A stock solution of 4-nitrophenyl acetate was prepared at 100 mM concentration in EtOH and stored at -20 °C. Rhodamine 6G chloride powder was kept at room temperature protected from direct light to avoid photobleaching.

Preparation of fluorophore-loaded plastics

PET-R6G, HPDE-R6G, PA6-R6G and PA66-R6G pellets were produced by melt blending of milled plastic granules with rhodamine 6G using a Euro Lab Prism 16 twin-screw extruder (Thermo Fisher Scientific, Waltham, MA, USA) with an L/D ratio of 25:1. The appropriate amounts of polymer and rhodamine 6G powders were mixed to achieve 1% w/w fluorophore loading before processing with an extruder at 500 RPM. The extrusion temperature varied for different resins. PE, PET, PA6, and PA66 were processed at 190°C, 250°C, 230°C, and 265°C, respectively. The extrudate strands were either air-cooled (PA6-R6G, PA66-R6G) or water-cooled (PE-R6G, PET-R6G) and then pelletized with an SGS 50-E pelletizer (C.F. Scheer & Cie, Stuttgart, Germany). The resulting pellets were stored at room temperature away from light. In total, 200 g of each substrate was prepared.

Prior to the experiments, additional washing was applied to remove fluorophore adsorbed on the surface of blended materials. The washing was performed by vortexing 4 g of pellets in 40 mL of milli-Q water or 100% EtOH for 5 minutes and discarding the liquid fractions. The number of wash steps and washing liquid depended on the blend type: the PET-R6G pellets were washed three times in Milli-Q water followed by one wash in 100% EtOH, the HDPE-R6G pellets were washed three times in 100% EtOH and the PA6-R6G and PA66-R6G pellets were washed five times in Milli-Q water, followed by two washes in 100% EtOH. The pellets were air-dried after the washing and stored at room temperature away from light until further use.

Determination of crystallinity in polymers

The crystallinity in virgin and blended materials was assessed by differential scanning calorimetry using a DSC250 calorimeter (TA instruments, New Castle, DE, USA). Plastic samples were encapsulated into sealed aluminium containers and subjected to heating according to the following protocol:

PET/PET-R6G: from 20 °C to 280 °C at a rate of 10°C/min followed by an isothermal hold of 5 min.

HDPE/HDPE-R6G: from 20 °C to 200 °C at a rate of 10°C/min followed by an isothermal hold of 5 min.

PA6/PA6-R6G: from 20 °C to 260 °C at a rate of 10°C/min followed by an isothermal hold of 5 min.

PA66/PA66-R6G: from 20 °C to 300 °C at a rate of 10°C/min followed by an isothermal hold of 5 min.

The percentage of crystallinity was calculated using the following equation:

$$Xc\ (\%) = \frac{\Delta H_m - \Delta H_{cc}}{\Delta H_f^{\ 0}} \times 100,$$

where ΔH_m is the observed enthalpy of melting, ΔH_{cc} is the observed enthalpy of cold crystallization and ΔH_f^0 is a reference value for the enthalpy of melting of 100% crystalline material (ΔH_f^0 values of 140 J g^{-1}, 293 J g^{-1}, 230 J g^{-1} and 226 J g^{-1} were used for PET, HDPE, nylon 6 and nylon 6,6, respectively [1]). The analysis was carried out in duplicates. Note that the cold crystallization term ΔH_{cc} was omitted when calculating the crystallinity of all plastics except for PET, as cold crystallization was observed for this polymer only.

Size exclusion chromatography (SEC) of polymer samples

Molar mass distributions of PET, nylon 6 and nylon 6,6 were assessed using a 1260 Infinity II LC system (Agilent Technologies, Santa Clara, CA, USA) equipped with three PL HFIPgel 250 x 4.6 mm columns (Agilent Technologies) and coupled to a multi-angle light scattering detector (miniDAWN TREOS; Wyatt Technology, Santa Barbara, CA, USA) and a differential refractive index detector (Optilab T-rEX; Wyatt Technology). Polymer samples were dissolved in 1,1,1,3,3,3-hexafluoroisopropanol supplied with 20 mM sodium trifluoroacetate (~5 mg/mL final polymer concentration) and analyzed as described previously [2].

Molar mass distributions of HDPE were assessed using an EcoSec HLC-8321 high temperature GPC system (Tosoh Bioscience LLC, Grove City, OH, USA) equipped with a differential refractive index (dRI) detector (Tosoh). Polymer separation was performed using three Tosoh TSK gel columns in the following order: TSK gel guard column (HHR (30) HT2 7.5 mm I.D. × 7.5 cm, PN 22891) and two sequential TSK gel GMHHR (20) HT2 (7.8 mm I.D. × 30 cm, PN 22888) columns. The mobile phase was 1,2,4-trichlorobenzene (TCB; Sigma Aldrich-HPLC Grade-P/N 256412) which was used as-received with no inhibitor added. Additionally, a reference column (TSK gel GMH HR-H (S) HT2; 7.8 mm I.D. × 30 cm) was employed to supply the eluent reference for the dRI detector. TCB was added to each sample to reach an end polymer concentration of ~1.4 mg/mL and heated at 160 °C on an external heating module for one hour with occasional agitation. The solvent stock was set to 40 °C while the pump oven was set to 50 °C. The columns, RI detector, injector valve, and autosampler were all set to 140 °C.

Samples were injected into a 300 µL sample loop and run at an operating flow rate of 1.0 mL/min for the sample columns. The reference column was set to an operating flow rate of 0.5 mL/min. Run time for all standards and samples was 40 minutes. A polystyrene-Quick Kit-M (Tosoh; PN 21916) was used to create a cubic polynomial calibration curve. Eco-Sec 8321 software (Tosoh) was used for data processing. Mark-Houwink correction values were applied to the polystyrene calibration curve [3]. Mark-Houwink values used for polystyrene were K = 12.1×10^{-5} dL/g and α = 0.707. Mark-Houwink values used for polyethylene were K = 40.6×10^{-5} dL/g and α = 0.725.

Protein expression and purification

Previously obtained plasmids encoding for LCC-ICCG [4] and $NylC_K$-TS [5] with C-terminal His-tags (pET-21(b)+ and pET-28(a)+, respectively) were used for protein expression in *E. coli*. The detailed information on these vectors has been previously deposited at the AddGene repository [https://www.addgene.org/Gregg_Beckham/].

Chemically competent BL21 Star (DE3) *E. coli* cells (Thermo Fisher Scientific; Waltham, MA, USA) were transformed with the corresponding plasmids according to the supplier's protocol. Single colonies were picked from LB agar plates containing 50 µg/mL kanamycin (pET-28(a)+) or 100 µg/mL ampicillin (pET-21(b)+) after overnight incubation at 37 °C and were further cultivated in LB medium overnight at the same temperature (200 RPM). These starter cultures were used to inoculate 0.5 L of LB medium supplied with appropriate antibiotics. The cells were then incubated at 37 °C, with shaking at 200 RPM, until an OD_{600} of approximately 0.6 was reached. Next, the cultures were cooled down to 25 °C and protein expression was induced by adding isopropyl thiogalactopyranoside (IPTG) to 0.5 mM final concentration. The cells were harvested by centrifugation (6,000 x g for 15 min) after 24 hours of growth and resuspended in 30 mL of binding buffer (50 mM Tris-HCl, pH 8.0, supplied with 5 mM imidazole and 500 mM NaCl). The cells in the resulting suspensions were lysed by sonication using a VibraCell ultrasonic disintegrator equipped with a micro tip probe (Sonics, Newtown, CT, USA) for 10 min in 5 s steps followed by 5 s pauses at 29% amplitude. The lysates were clarified by centrifugation at 20,000 x g for 15 min at 4 °C and filtered through a 0.22 µm syringe filter. The enzymes were purified from lysates by immobilized metal affinity chromatography using a 5 mL Ni-charged HisTrap FF column (Cytiva, Marlborough, MA, USA). The proteins were eluted with a linear gradient of imidazole (5 – 500 mM) in binding buffer (20 column volumes at 2.5 mL/min flow rate). Protein preparations were analyzed with SDS-PAGE. Imidazole and NaCl were removed from enzyme stock solutions by multiple rounds of concentration-dilution using 50 mM Tris-HCl buffer (pH 8.0) and Vivaspin ultrafiltration tubes (10 kDa MWCO; Sartorius, Göttingen, Germany). Protein concentrations were determined by UV

spectroscopy (λ = 280 nm) using theoretical extinction coefficients calculated with ProtParam tool [https://web.expasy.org/protparam/].

Detection of PETase activity in *E. coli* lysates and culture medium

Chemically competent BL21 Star (DE3) *E. coli* cells (Thermo Fisher Scientific; Waltham, MA, USA) were transformed with a pET-21(b)+ vector encoding for LCC-ICCG, spread on an LB agar plate containing 100 µg/mL ampicillin and incubated overnight at 37 °C. A single colony was transferred into 20 mL of fresh LB medium supplied with the same antibiotic at the same concentration. The cells were incubated overnight at 37 °C, 200 RPM. To generate a control culture, untransformed BL21 Star (DE3) *E. coli* cells were grown overnight in 20 mL of LB medium (37 °C, 200 RPM) in the absence of antibiotic. Next, both cultures were induced by adding IPTG to 1 mM final concentration and further incubated for 3 more hours at 37 °C and 200 RPM. Cells were harvested by centrifugation at 4,000 x g for 15 minutes, resuspended in 20 mL of 50 mM Tris-HCl, pH 8.0, and subjected to ultrasonic lysis as described above (see "Protein expression and purification"). Half of the volume of each lysate was clarified by centrifuging at 20,000 x g for 15 minutes, whereas the other half remained unprocessed. 50 µL of crude lysates, clarified lysates and culture medium were diluted 10-fold with 50 mM Tris-HCl, pH 8.0, and co-incubated with PET-R6G (1 pellet per reaction) at 60°C, 500 RPM for 64 hours before measuring fluorescence.

Inhibition of LCC-ICCG by rhodamine 6G

LCC-ICCG inhibition by rhodamine 6G was assessed using 4-nitrophenyl acetate as a model substrate. Reaction mixtures (1 mM 4-nitrophenyl acetate in 50 mM Tris-HCl, pH 8.0) containing 0, 10, 100 or 1000 µg/mL fluorophore were loaded into a 96-well microtiter plate (100 µL per well). The experiments were initiated by adding enzyme to 0.1 µM final concentration or by adding the same volume of milli-Q water. The hydrolysis of 4-nitrophenyl acetate was followed at the room temperature by measuring the absorbance of 4-nitrophenol at 400 nm using a VarioSkan Lux plate reader (Thermo Fisher Scientific; Waltham, MA, USA). The reaction rates (mAU * min^{-1}) were determined using linear parts of the resulting progress curves.

Rhodamine 6G toxicity assay

BL21 Star (DE3) *E. coli* cells were cultivated overnight at 37 °C, 200 RPM in 20 mL LB medium and diluted to OD_{600} = 0.01 using a fresh batch of the same medium. 100 µL aliquots were transferred from the diluted culture to a 96-well microtiter plate (1 aliquot per well). To each well, 80 µL of fresh LB medium was added followed by 20 µL of aqueous rhodamine 6G solutions (or Milli-Q water) to yield a final fluorophore concentration of 0, 10 or 100 µg/mL (3 replicates for each fluorophore

concentration). The plate was sealed with a transparent film, which was punctured with a syringe needle on top of each well once to allow for gas exchange. The cell growth experiment was carried out at 37°C for 33 h in a Varioskan Lux plate reader (Thermo Fisher Scientific; Waltham, MA, USA), with shaking at 180 RPM for 10 seconds every 5 minutes and OD_{600} recording every 10 min.

Passive diffusion of rhodamine 6G from various plastics

For each type of blended polymer material, 1 pellet was transferred into a 1.5 mL microcentrifuge tube containing 1 mL of 50 mM Tris-HCl, pH 8.0, and incubated for 23 hours at 65 °C, 1000 RPM. 20 µL aliquots were taken at different time points and diluted 10-fold with the same buffer in a 96-well non-transparent microtiter plate prior to measuring the fluorescence using a Varioskan Lux plate reader. The experiments were carried out in triplicates.

Supplementary references

1. TA Instruments. Thermal applications note. Polymer heats of fusion; https://www.tainstruments.com/pdf/literature/TN048.pdf.
2. Cuthbertson, A. A., Lincoln, C., Miscall, J., Stanley, L. M., Maurya, A. K., Asundi, A. S., Tassone, C. J., Rorrer, N. A. & Beckham, G. T. (2024) Characterization of polymer properties and identification of additives in commercially available research plastics, *Green Chemistry.* **26**, 7067-7090.
3. Agilent Technologies. Repeatability in high temperature polyethylene analysis using Agilent PLgel Mixed-B; https://www.agilent.com/cs/library/technicaloverviews/public/5990-8496EN.pdf?srsltid=AfmBOopPI2rW3FkwV_1IUjTCjGs9sjEWz4Q7iBziZAAjNBcZmmxRAtfl.
4. Tournier, V., Topham, C. M., Gilles, A., David, B., Folgoas, C., Moya-Leclair, E., Kamionka, E., Desrousseaux, M. L., Texier, H., Gavalda, S., Cot, M., Guémard, E., Dalibey, M., Nomme, J., Cioci, G., Barbe, S., Chateau, M., André, I., Duquesne, S. & Marty, A. (2020) An engineered PET depolymerase to break down and recycle plastic bottles, *Nature.* **580**, 216-219.
5. Bell, E. L., Rosetto, G., Ingraham, M. A., Ramirez, K. J., Lincoln, C., Clarke, R. W., Gado, J. E., Lilly, J. L., Kucharzyk, K. H., Erickson, E. & Beckham, G. T. (2024) Natural diversity screening, assay development, and characterization of nylon-6 enzymatic depolymerization, *Nat Commun.* **15**, 1217.
